# Development of *In Vitro*-*In Vivo* Correlation/Relationship Modeling Approaches for Immediate Release Formulations Using Compartmental Dynamic Dissolution Data from “Golem”: A Novel Apparatus

**DOI:** 10.1155/2015/328628

**Published:** 2015-05-18

**Authors:** Martin Čulen, Paweł K. Tuszyński, Sebastian Polak, Renata Jachowicz, Aleksander Mendyk, Jiří Dohnal

**Affiliations:** ^1^Department of Chemical Drugs, Faculty of Pharmacy, University of Veterinary and Pharmaceutical Sciences Brno, Palackého 1-3, 612 42 Brno, Czech Republic; ^2^Department of Internal Medicine, Hematology and Oncology, Faculty of Medicine, Masaryk University, Kamenice 5, 625 00 Brno, Czech Republic; ^3^Department of Pharmaceutical Technology and Biopharmaceutics, Faculty of Pharmacy, Jagiellonian University Medical College, Medyczna 9 Street, 30-688 Kraków, Poland; ^4^Department of Pharmacoepidemiology and Pharmacoeconomics and Department of Social Pharmacy, Faculty of Pharmacy, Jagiellonian University Medical College, Medyczna 9 Street, 30-688 Kraków, Poland; ^5^Simcyp (a Certara Company) Limited, Blades Enterprise Centre, John Street, Sheffield S2 4SU, UK; ^6^Department of Applied Pharmacy, Faculty of Pharmacy, University of Veterinary and Pharmaceutical Sciences Brno, Palackého 1-3, 612 42 Brno, Czech Republic

## Abstract

Different batches of atorvastatin, represented by two immediate release formulation designs, were studied using a novel dynamic dissolution apparatus, simulating stomach and small intestine. A universal dissolution method was employed which simulated the physiology of human gastrointestinal tract, including the precise chyme transit behavior and biorelevant conditions. The multicompartmental dissolution data allowed direct observation and qualitative discrimination of the differences resulting from highly pH dependent dissolution behavior of the tested batches. Further evaluation of results was performed using IVIVC/IVIVR development. While satisfactory correlation could not be achieved using a conventional deconvolution based-model, promising results were obtained through the use of a nonconventional approach exploiting the complex compartmental dissolution data.

## 1. Introduction

An orally administered drug has to be released from its dosage form, dissolved in the surrounding fluid and absorbed by the gut wall, in order to enter the blood stream. Dissolution testing in pharmacy studies the first two processes and is not only a vital tool for assessment of quality of a pharmaceutical, but also a tool for elucidation and simulation of these effects* in vitro*. The research in drug development facilitates dissolution to uncover and predict many crucial aspects influencing the fate of an administered active pharmaceutical ingredient (API) in the gastrointestinal tract (GIT), while employing a wide variety of innovative and special apparatuses, either based on the conventional pharmacopeial tools or having a completely original design [1]. Such instruments usually involve only one or two compartments, and they functionally often address only a specific focus of a study, that is, drug precipitation, mechanical qualities of a dosage form, and so forth [2]. However, a more complex simulation of the GIT is often needed, and currently only TIM-1 apparatus fully enables* in vitro* testing in completely biorelevant conditions ranging from stomach to ileum [3].

In order to provide other means of highly biorelevant and dynamic dissolution testing, our team has developed a novel four-compartmental dissolution apparatus, named “Golem,” which simulates the dissolution processes in stomach and small intestine (SI). This paper discusses the application and evaluation of this apparatus, performed with several generic and reference batches of immediate release tablets containing atorvastatin (ATV). This drug was chosen due to the availability of* in vivo* data from subsequent bioequivalence (BE) studies for the tested formulations.

ATV is characterized by low solubility in water and high permeability and is hence classified as a Class II drug in the Biopharmaceutical Classification System (BCS) [4]. Dissolution is the rate-limiting step in absorption of ATV, and IVIVC could be expected according to the BCS theory. But being a drug of limited bioavailability, due to high variability in first-pass metabolism, makes it difficult to correlate the conventional* in vitro* tests results with blood concentration in time (or other pharmacokinetic parameters). Therefore an attempt to establish an* in vitro*-*in vivo* correlation/relationship model for this drug was made, based on the dissolution data obtained from this novel unconventional dissolution instrument.

## 2. Materials and Methods

### 2.1. Golem Apparatus

**Figure 1:**
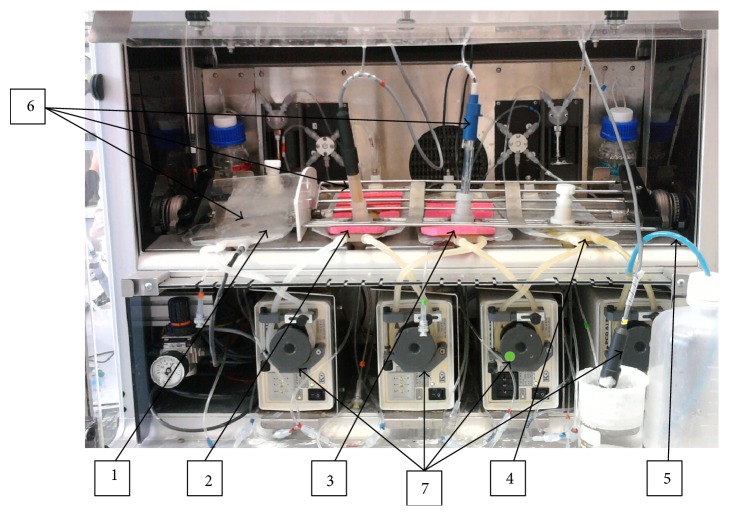
Front view with description of main components: (1) stomach, (2) duodenum, (3) jejunum, (4) ileum, (5) collection canister tubing, (6) pH probes, and (7) peristaltic pumps.

The instrument is a computer controlled artificial digestive tract, designed for dynamic dissolution testing of oral dosage forms and consisting of four compartments: stomach, duodenum, jejunum, and ileum (see Figure 1). Physiological conditions are maintained in the system with the possibility of adjusting all method parameters, for example, pH, volumes, transit times, temperature, and so forth. The dissolution in fed state can be tested with liquid meal (e.g., Nutridrink, Ensure Plus, or very finely homogenized solid meal to prevent clogging of the pump tubes). The transport of chyme in Golem is driven by peristaltic pumps placed between each two subsequent compartments, where the fourth pump leads the chyme from the ileum into the collection canister (waste). The pH in the compartments is checked automatically by pH probes and is manually adjusted by injection of either 1 M NaOH + 0.24 M NaHCO_3_ or 1 M HCl solution.

The compartments are made from modified common intravenous bags (the plastic was tested for interaction with various APIs). The bags involve three ports: one holds plastic tubes for injection of pH altering solutions, enzymes, and sample collection; second port serves as a mouthpiece for the pH probe, and the third port is used for tablet insertion into the compartments. Temperature is kept at physiological 37°C and continuously checked separately for: (a) the heater platform; (b) the stomach compartment; (c) the air inside the apparatus box. Peristaltic movement is simulated by a V-shaped grate, pressed down on the bags, which rocks from side to side, driven by compressed air.

The operation of the apparatus requires one person for manual collection of samples and injection of enzymes, using common syringes.

From perspective of biorelevancy, the Golem apparatus is one of the three most complex dissolution apparatuses described to date (other two being TIM-1 and Dynamic Gastric Model) [3]. Its main advantage is that the complex functions are based on simple technical solutions which make the apparatus user friendly and easy to modify, and at the same time significantly less expensive than the alternatives.

The instrument was designed by Řezáčová, Dohnal, Jampílek and Čulen, and constructed by Development Workshops of the Institute of Organic Chemistry and Biochemistry of the Czech Academy of Sciences [5].

### 2.2. Dissolution Method

A method simulating fasted state and developed according to previous work was used in the study [6].

**Figure 2:**
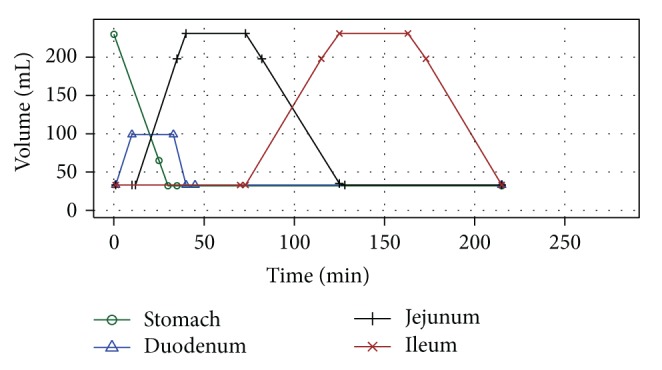
An approximated visual scheme of chyme transit in the Golem apparatus. A residual volume equal to the starting volume remained in each compartment, therefore the “total” amount moved through the apparatus was 200 mL.

Prior to the dissolution experiments, the contents of the compartments were heated to 37°C. The experiments were started directly after insertion of a dosage form into the first stomach compartment. The gastric medium containing both the dissolved and undissolved contents of the dosage form was gradually moved by the peristaltic pump from stomach into the duodenal compartment and then into the following two compartments (an approximate scheme of chyme transfer in Golem is depicted in Figure 2). The medium thus underwent a dynamic change as it merged with the contents (starting volumes) of the next compartments. The starting dissolution medium was based on a physiological solution with pH modified by hydrochloric acid or bicarbonate buffer; pepsin was added only to the gastric compartment. The pH values, concentration of bile salts (bile extract porcine, Sigma-Aldrich), and lipase (pancreatin, Zentiva) in the SI compartments were maintained at steady levels throughout the experiment. The pH in stomach compartment was left to change, being influenced by the studied formulation. The detailed information on the method design is given in Table 1.

All experiments were performed in duplicates.

### 2.3. Sample Analysis

The samples were collected manualy using common syringe; 1 mL of sample was filtered through a 0.25 *μ*m syringe filter, and the rest was returned into the apparatus. The withdrawal of the medium and dissolved API was considered in the calculations. The filtered sample was analyzed with HPLC (Waters) at *λ* = 246 nm.

### 2.4. Formulations Tested

In total, five generic (Zentiva) and three reference originator immediate release tablet batches were tested with Golem, all containing 80 mg of atorvastatin, present as a calcium salt. From the generic batches, four contained amorphous form of the drug (batches 85, 82, 01, and 02), which had higher intrinsic dissolution rate compared with the crystal form contained in the generic batch80 and all the reference batches (Lipitor, Sortis06, Sortis10) [10]. All formulations with the crystal ATV also contained CaCO_3_ as a buffering agent used to raise the gastric pH and thus facilitate very early dissolution of ATV, which is a weak acid almost insoluble in pH below 4. The amorphous generic batches, on the other hand, contained no buffer, which was to slow down the faster dissolution of the amorphous form of the drug.

### 2.5. Pharmacokinetic (PK) Studies

The BE studies of the five generic formulations were evaluated in four crossover PK studies, performed by other contract workplaces: (a) Batch85 versus Sortis03, 24 subjects; (b) batches 01 and 02 versus Sortis03, 24 subjects; (c) batch82 versus Sortis10, 102 subjects; (d) batch80 versus Lipitor, 81 subjects.

### 2.6. Modeling

In order to establish a relationship between dissolution test results and actual results from* in vivo* studies, different modeling approaches were applied.

#### 2.6.1. Classical Approach

A conventional method was based on a numeric deconvolution, where whole* in vitro* profiles of cumulative fraction dissolved were taken into account, with or without exclusion of the measurements from selected compartments. The *R* statistical computing software was used for modeling. An  Rivivc package [11] and self-written *R* scripts were used to preprocess data and perform numerical deconvolution. In order to calculate fraction of drug absorbed in time, simulated* in vivo* profile (*i.v.* administration) was used as an equivalent to the unit impulse response (UIR). Simulation of* in vivo* profile after* i.v.* administration was carried out on Simcyp Population-based Simulator V13R1 and was based on the results for ten virtual healthy patients [12]. Minimal PBPK model with single adjusting compartment (SAC) was utilized. Volume of distribution was calculated with use of the model based upon a modified version of the Poulin and Theil method [13]. Input information covered system data specific for the chosen population (as provided by simulator), trial design information (single 30 seconds long* i.v.* bolus, 80 mg), and compound specific data. Physicochemical, binding, and ADME (absorption, distribution, metabolism, and excretion) data are presented in Table 2. Renal clearance of ATV was assumed to be negligible [14].

#### 2.6.2. Compartmental Approach

A second approach was based on direct scaling of the outcome of tests carried on Golem apparatus, where particular compartments were correlated with* in vivo* profiles.

### 2.7. Model's Predictability Evaluation

A proper IVIVC model should be established using formulations with different release rates. At minimum three (slow, medium, and fast dissolving) dosage forms should be designed, where the extreme ones are used to build a model able to predict* in vivo* bioperformance of the “middle” formulation. Such an approach is common when sustained-release dosage forms are tested but becomes problematic with immediate-release formulations (IR). All batches of the model drug, atorvastatin, were of immediate release and in the case of either the buffered or the nonbuffered formulations were behaving similarly. Therefore, external predictability and validation were evaluated when one formulation was characterized by medium rate release kinetics, used as testing formulation, and two other formulations were used for model building. In case of internal validation, testing formulation was included in model building phase. Whole or partial area under the curve (pAUC) prediction error (PE) (1) and *C*_max⁡_ prediction error (2) was calculated [15]:

(1) $$\begin{array}{c} \text{pAUC}\_\text{PE}\left[\%\right]=\left[\frac{\left|{\text{pAUC}}_{\text{observed}}-{\text{pAUC}}_{\text{predicted}}\right|}{{\text{pAUC}}_{\text{observed}}}\right]*100, \end{array}$$

(2) $$\begin{array}{c} C_{\max}\_\text{PE}\left[\%\right]=\left[\frac{\left|C_{\max,\text{observed}}-C_{\max,\text{predicted}}\right|}{C_{\max,\text{observed}}}\right]*100. \end{array}$$

## 3. Results and Discussion

### 3.1. PK Studies

Bioavailability of the five generic batches was compared with three batches of the reference product (atorvastatin 80 mg manufactured by Pfizer, under the brand names Lipitor and Sortis, sold on different markets), in total of four consecutive PK studies. The PK data served as a qualitative “ladder” for the dissolution experiments and as basis for IVIV correlations.

**Figure 3:**
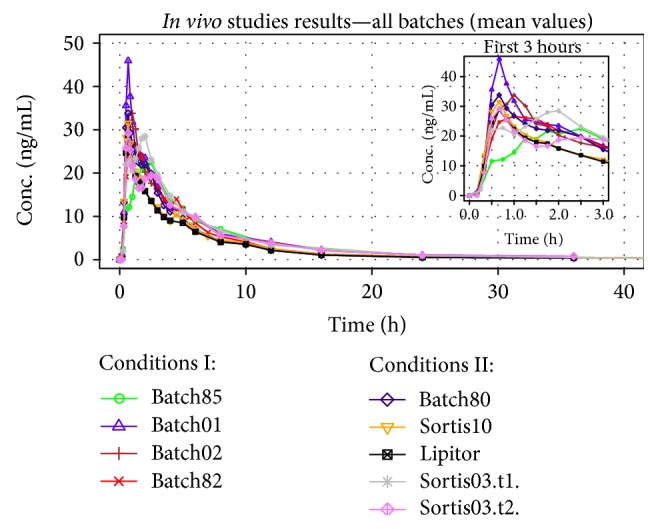
Bioequivalence studies results. Sortis03 was tested twice, in two PK studies.

The particular BE study results were as follows: (a) generic batch85 compared with Sortis03 showed *C*_max⁡_ below the approved range for bioavailability (90% confidence interval (CI) ratio of 75%); (b) both batches 01 and 02 later showed much higher *C*_max⁡_ than the reference Sortis03 (90% CI ratio 160 and 140%, resp.); (c) the batch82 was bioequivalent to Sortis10; (d) the much later developed batch80 (first generic batch with crystal API) has shown slightly higher but unacceptable bioavailability, with upper 90% CI of 127%. The average plasma concentration-time profiles are plotted together in Figure 3.

In general, the drug's pharmacokinetics showed a considerable interindividual variability, which is well reflected in the average PK profiles (see Figure 2). The most important origin of the variability seems to be the gut and liver metabolism of the drug [16]. An important observation from the average PK curves is the very short *T*_max⁡_ values (0.67–1.00 h), which suggested a very rapid dissolution and absorption in GIT.

### 3.2. Dissolution Results

**Figure 4:**
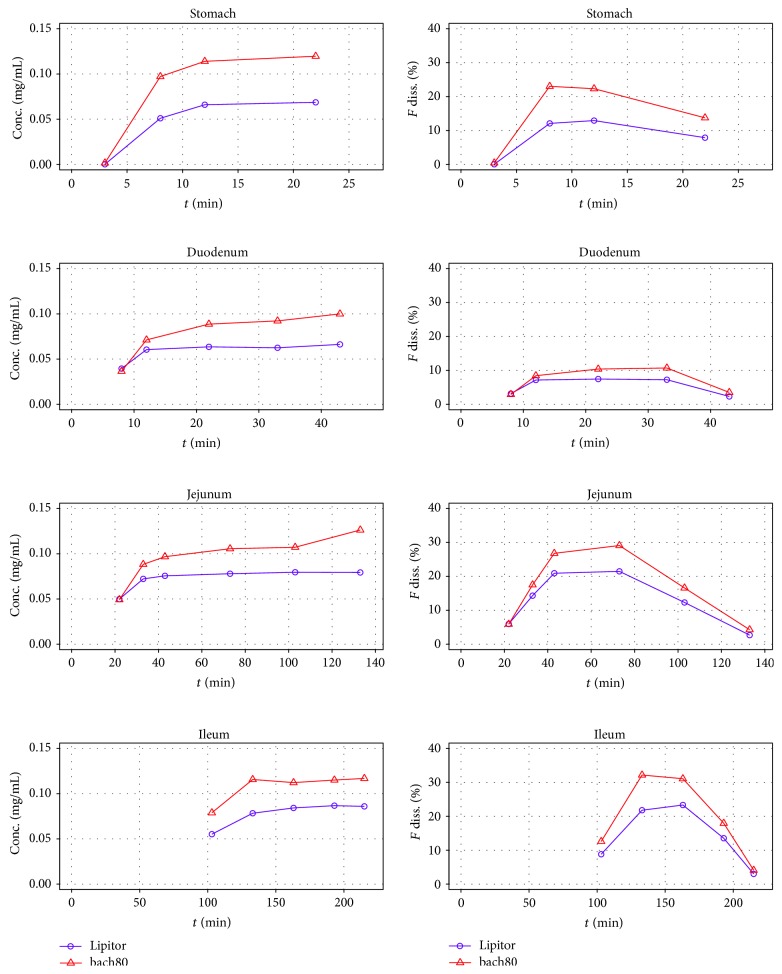
Golem dissolution results for nonbuffered generic batch80 and its reference product Lipitor. The plots show dissolution behavior measured in separate compartments, as well as cumulative profile showing the amount of drug dissolved in the whole apparatus including the amount gradually transferred from ileum into the collection canister. The terminal decrease of dissolved amount in individual compartments was caused by transfer of portions of the medium into the following compartment, or into the collection canister in case of ileum.

The Golem dissolution experiments were started with batch80 and its reference product Lipitor, since both products contained the same crystal form of ATV, and both were buffered by CaCO_3_, as excipient. The two formulations were run with the full-length fasted state dissolution method, lasting 215 min.  Figure 4 shows the dissolution results as concentration or fraction of drug dissolved for all four Golem's compartments separately and also in a cumulative profile. Both formulations showed almost parallel dissolution profiles, but with higher dissolved amount in case of batch80, which qualitatively corresponded with the* in vivo* results. Generally, the tablets were observed to disintegrate in the first 3 min; the API quickly dissolved and the slight increase in the cumulative dissolved amount of API after 100 min could be mainly accounted for the higher pH in ileum. The fact that the fraction dissolved did not surpass 45% was most probably caused by the salting out effect of the Ca^2+^ counter-ion coming from the buffering excipient.

Since the maximum plasma concentrations* in vivo* were reached between 40 and 60 min and the dissolution profiles of the IR tablets provided little information at later time points, all further dissolution tests with the remaining batches were decided to be run with the same fasted state method but terminated directly after the sampling point at 43 min.

**Figure 5:**
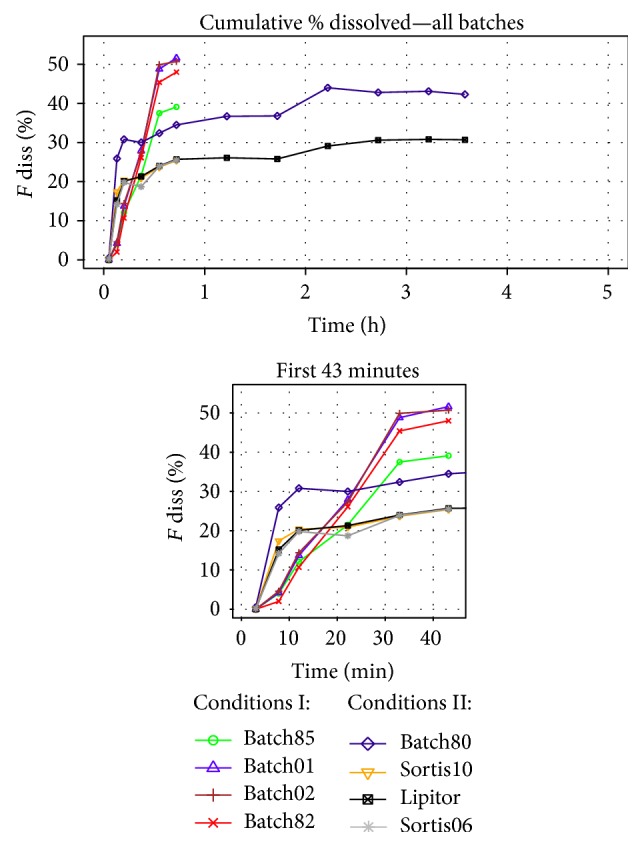
Cumulative fraction of ATV dissolved in whole apparatus.

The further tested buffered formulations included two reference batches from the BE studies (Lipitor and Sortis10), plus one other batch of the reference product, Sortis06. All three reference batches showed almost identical dissolution behavior (see Figure 5), which confirmed that they were interchangeable in the dissolution experiments.

The dissolution testing of nonbuffered generic formulations revealed that no API dissolved in the stomach compartment, although the tablets disintegrated completely in less than 3 min. In contrast, all buffered formulations, generic and reference alike, raised the gastric pH to 7-8, which enabled rapid dissolution of ATV. The results came as a surprise in comparison with traditional USP II tests, which were performed with 900 mL of simple buffered media, where the pH was raised by the buffered formulations only to values around 4, thus prohibiting discrimination between the two formulation designs (unpublished results). This observed difference between the buffered and nonbuffered formulation in Golem provided a useful hint on the manner of* in vivo* dissolution behavior.

As for the nonbuffered batches, these generally provided higher fraction dissolved as they did not contain the CaCO_3_ buffering agent. Other than that, the batch85 with low bioavailability* in vivo* had the lowest dissolution performance, followed by the bioequivalent batch82, and then the two batches with highest bioavailability—batch01 and batch02. However, the dissolution profiles for batches 01 and 02 were identical, and, according to similarity and difference factor, they also did not differ from the profile of batch82. Moreover, API powder which served as a control also showed identical dissolution performance to batches 01 and 02, indicating that the dissolution method lacked discriminatory power for these highly dissolving batches. This was caused by low medium volume (although physiological) in combination with lack of an absorption step (sink-condition). Nevertheless, the dissolution method was developed as a universal, most physiologically relevant simulation of fasted state, and as such it provided results beyond expectation. The obvious and simple option for improvement of dissolution testing of drugs with low solubility (such as atorvastatin) would be modification of the method by employing higher (unphysiological) medium volumes in the individual compartments, which would prevent early saturation of medium with the tested API. Of course, certain very lipophilic drugs would require unachievably high medium volumes to enable full dissolution of the administered dose. It is however questionable whether this is necessary when considering the fact that such drugs would neither be expected to fully dissolve* in vivo*. However, study of these effects was beyond the scope of the present study.

### 3.3. IVIVC-Classical Approach

The results depicted below follow a general agenda of* in vitro*-*in vivo* correlation level A, where fraction absorbed* in vivo* is directly correlated to the fraction dissolved* in vitro*. A linear function is preferred for this correlation, although other reasonable mathematical relationships are allowed when properly justified. The key procedure here was a deconvolution of a PK profile into the cumulative curve describing fraction absorbed* in vivo*. Since atorvastatin PK p.o. profile does not follow 1-compartment nor even 2-compartment model, classical Wagner-Nelson or Loo-Riegelman deconvolution methods could not be applied here. Instead, more sophisticated numerical deconvolution was employed. Numerical deconvolution relies on the availability of a PK profile of drug administered intravenously (*i.v.*), thus representing pure distribution/elimination phase, without absorption. Since in the presented case, bioassay protocol did not include such atorvastatin administration, an approximation of* i.v.* profile by computer simulations, carried out with Simcyp software, was used.

**Figure 6:**
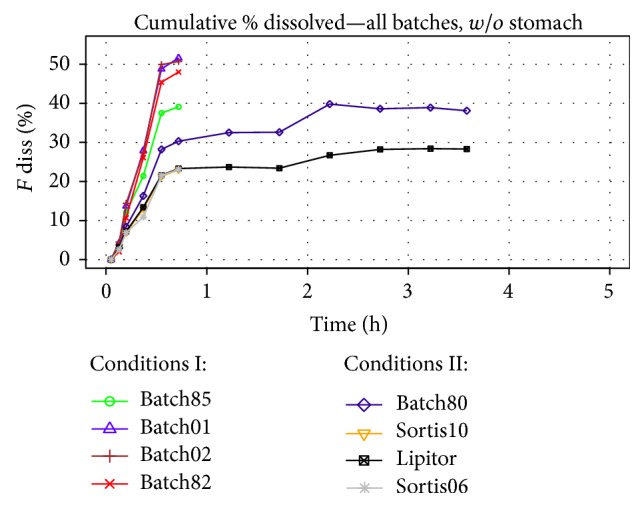
Cumulative fraction of ATV dissolved in duodenum, jejunum, and ileum compartments.

In case of the dissolution results available, the presence or absence of the buffering agent affected whether drug did dissolve in the stomach compartment, which produced two distinct cumulative profile patterns and significantly limited the comparison of the two formulation designs.* In vivo* absorption of atorvastatin is believed to start only in small intestine and thus a deconvolution of plasmatic profile would yield a fraction of drug absorbed only from small intestine. According to this, the fraction of drug dissolved in stomach compartment was excluded from the cumulative profiles used for the IVIV correlations. The final profiles used for correlation are depicted in Figure 6.

**Figure 7:**
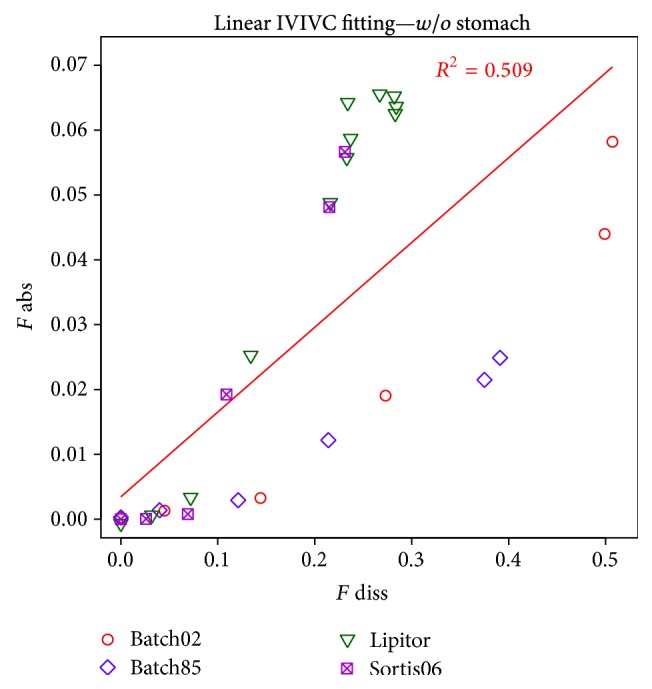
IVIVC plot for batches tested in two different conditions.

**Figure 8:**
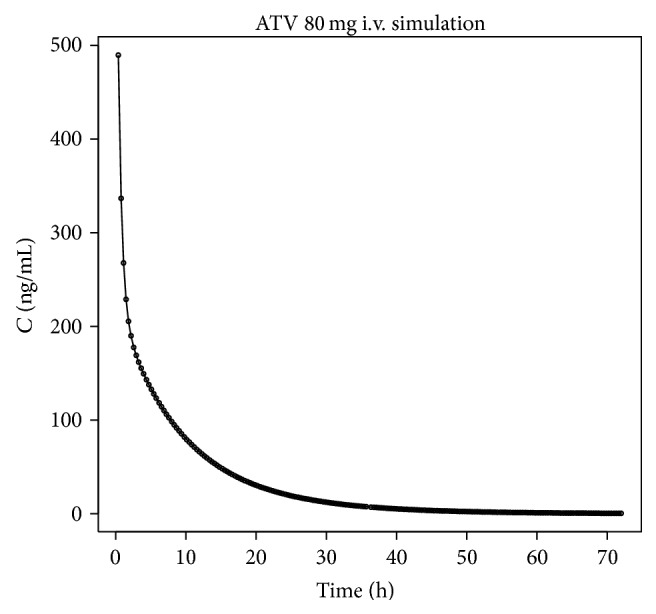
Simulated* in vivo* profile after administration of 80 mg of ATV.

Despite the previous modification of the profiles, the lower solubility of ATV in the presence of CaCO_3_ and the resulting overall lower dissolution performance of the buffered batches caused that it was difficult to build a reliable model using combination of buffered and nonbuffered formulations. Therefore, models had to be built for Design I (buffered batches) and Design II (nonbuffered) separately.  Figure 7 depicts deconvolution results for two formulations of both designs and the lack satisfactory level of correlation (*R* ^2^ = 0.37). Deconvolution was performed using simulated* in vivo* response after intravenous administration of 80 mg of ATV (Figure 8).

*In vitro*-*in vivo* correlation was described by both linear and nonlinear (polynomial) relationship (3). The lm() *R* base function was used:

(3) $$\begin{array}{c} \text{FABS}=B_{0}+B_{1}*\text{FDISS}+B_{2}*{\text{FDISS}}^{2}+E, \end{array}$$

where FABS is the fraction absorbed, *B*_0_ is the *y*-intercept, *B*_1_ and *B*_2_ are coefficients, FDISS is the fraction dissolved, and *E* are residuals.

#### 3.3.1. Design I

**Figure 9:**
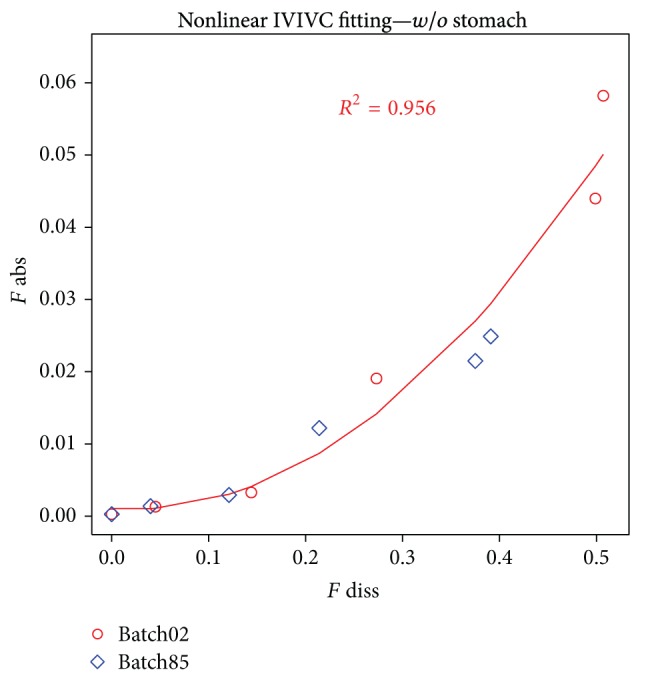
Deconvolution results for nonbuffered batch02 and batch85.

In the nonbuffered group a model was built based on batch01 and batch85 and nonlinear (polynomial) correlation was obtained with *R* ^2^ = 0.951 (Figure 9).

**Figure 10:**
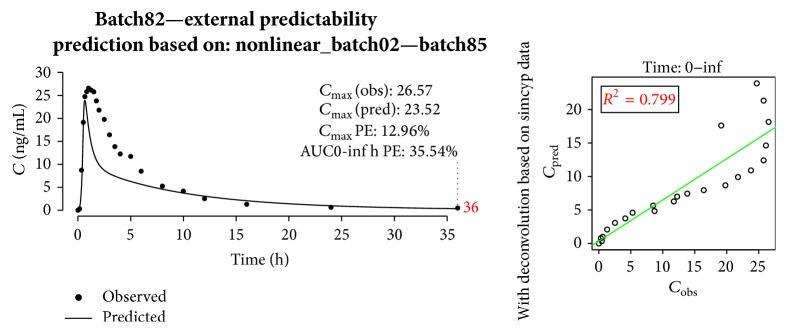
Prediction of* in vivo* profile of batch82 with nonlinear model built on batch02 and batch85.

Predictability evaluation was performed by calculating fraction of ATV absorbed versus time with regression equation with* in vitro* profile as an input. With intravenous response profile, numerical convolution was performed, and the prediction of* in vivo* curve was obtained.  Figure 10 depicts simulation of* in vivo* profile of batch82.

The model's prediction errors for *C*_max⁡_ and AUC did not meet the FDA criteria [15] and were to be rejected.

#### 3.3.2. Design II

**Figure 11:**
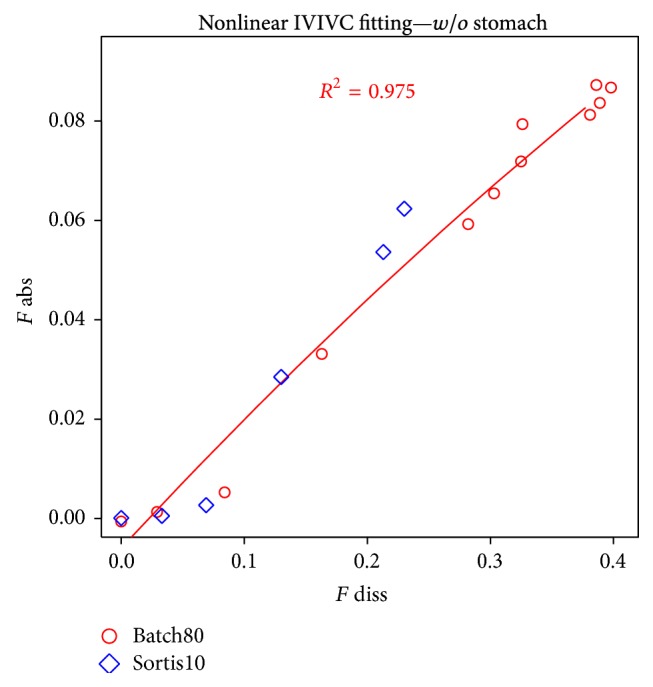
Nonlinear IVIVC example.

**Figure 12:**
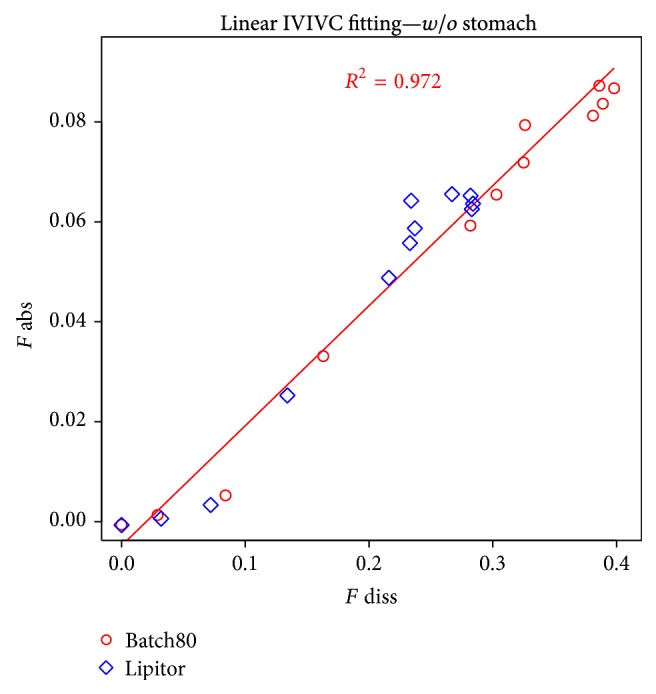
Linear IVIVC example.

Deconvolution results for buffered batches are presented in Figures 11 and 12. In most cases, nonlinear correlation was observed.

**Figure 13:**
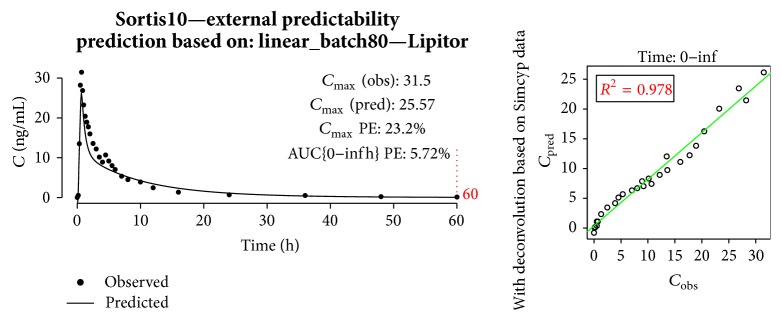
Prediction of Sortis10 profile.

In Figure 13, prediction of Sortis10 plasma concentration is shown. Although AUC PE was low (5.75%), the prediction of *C*_max⁡_ was not satisfactory.

**Figure 14:**
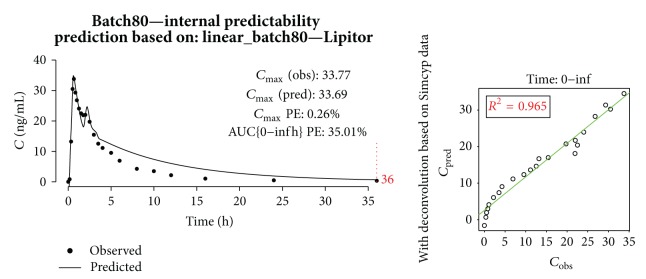
Prediction of generic batch80 profile with linear model built on batch80 and Lipitor.

An example of internal predictability is presented in Figure 14. In general, predictions of *C*_max⁡_ were good, but the descending curves could not properly reflect the* in vivo* behavior resulting in high AUC PE. This was partially due to the fact that the simulated* i.v.* profile used for deconvolution (and later convolution in the validation phase) was the same for all batches and not fitted to each group of patients participating in clinical trials.

### 3.4. IVIVC/IVIVR-Compartmental Approach

In order to fully exploit Golem's features, it was attempted to create IVIVR introducing completely nonstandard approaches, where no conventional convolution/deconvolution methods are necessary. This implies no more requirements for* i.v.* administration results, and therefore results obtained in a more cost-saving manner, utilizing the Golem data for IVIVC/IVIVR development.

**Figure 15:**
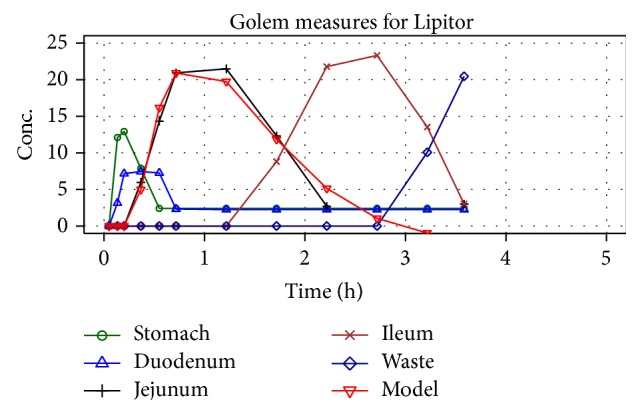
Example of measurements performed with Golem apparatus (for Lipitor).

The Golem apparatus allows measuring the concentration of API in each compartment separately. An example of distribution of dissolved API for Lipitor is depicted in Figure 15.

**Figure 16:**
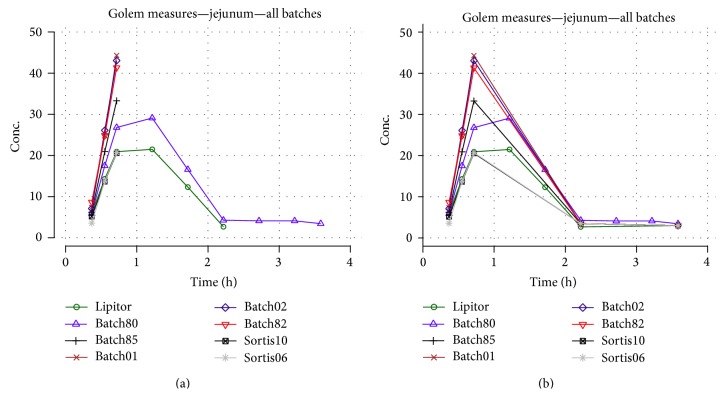
Dissolution in jejunum compartment (a) and addition of two additional time points (b).

Various approaches were examined to correlate results for each compartment with corresponding* in vivo* profiles, but successful outcome was reached only with jejunum compartment. As shown in Figure 16, profiles were described by nonlinear function:

(4) $$\begin{array}{c} f\left(t\right)=A*e^{\left(-k1*t\right)}+B*e^{\left(-k2*t\right)}+C*e^{\left(-k3*t\right)}, \end{array}$$

where *f*(*t*) is the concentration of an API at given sampling time *t*; *A*, *B*, and *C* are the equation constants; *k*1, *k*2, and *k*3 are constants.

The model presented above follows a general equation for 2-compartment model with p.o. administration, and despite the previously mentioned claim that ATV PK profile does not follow such model well, it was chosen as a compromise between modeling accuracy and optimization procedure stability.

Formula was optimized with nonlinear Solver (DEPS: differential evolution and particle swarm optimization) available in LibreOffice Calc (ver. 4.1.5.3). Figure 16 represents measurements for jejunum compartment for each batch (Figure 16(a)). A complete measurement was available only for Lipitor and batch80, and the descending part of the* in vitro* curves for the six remaining batches had to be introduced by addition of two time points (Figure 16(b)) in order to perform* in vivo* time scaling and correlation. Correlation of full profiles was performed for Lipitor and batch80 and was used for internal validation of the model. Only the ascending (and* in vitro* measured) parts of profiles were correlated for the remaining batches.

**Figure 17:**
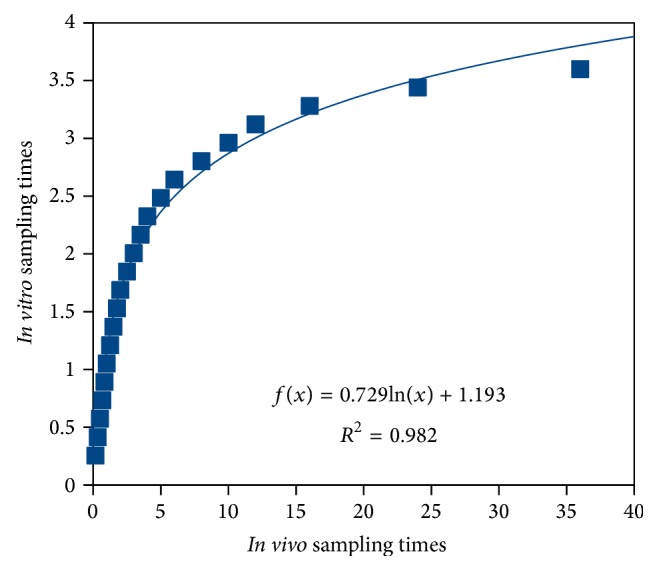
Logarithmic scaling of* in vivo* profile.

As shown in Figure 17,* in vivo* time scale was scaled nonlinearly, however with a simple reversible mathematical function.

Finally, with (5), predicted plasma concentrations were calculated for all batches. Two constants were introduced for linear scaling of* in vitro* profile and were optimized with nonlinear Solver:

(5) f(t)=CONST1∗[A∗e(−k1∗t)+B∗e(−k2∗t)+C∗e(−k3∗t)] +CONST2,

where *f*(*t*) is the predicted plasma concentration at given time *t*; *A*, *B*, *C*, *k*1, *k*2, and *k*3 are constants calculated for* in vitro* jejunum profile (4); CONST1 and CONST2 are constants for whole* in vitro* model linear scaling.

For CONST1 and CONST2 optimization, only two formulations were used and a middle one (with medium dissolution rate) was predicted.

#### 3.4.1. Designs I and II

**Figure 18:**
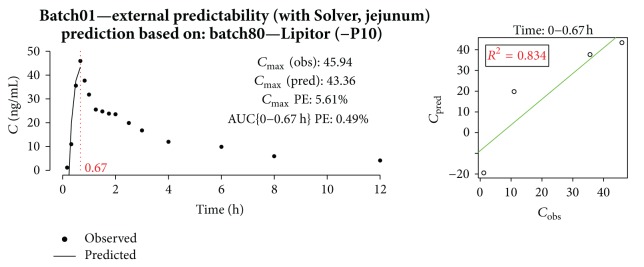
Prediction of batch01 profile with model built on batch80 and Lipitor.

Results for external prediction of batch01 (amorphous ATV, nonbuffered) PK profile are presented in Figure 18. Although constants for* in vitro* profile scaling were optimized for batch80 and Lipitor (buffered conditions), pAUC PE and *C*_max⁡_ PE for batch01 were below 10%.

#### 3.4.2. Design II

**Figure 19:**
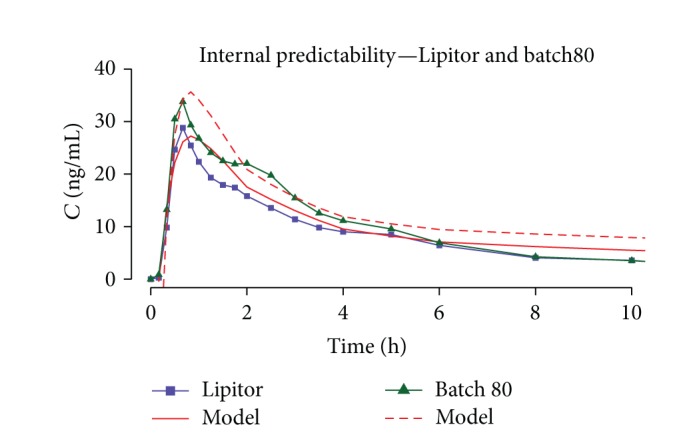
Comparison of Lipitor and batch80 predicted and actual plasma concentration profiles.

Model was optimized for batch80 and Lipitor, since these were the two most extensively studied batches (215 min* in vitro* profiles), and their* in vivo* profiles sufficiently differed between each other (Figure 19).

**Figure 20:**
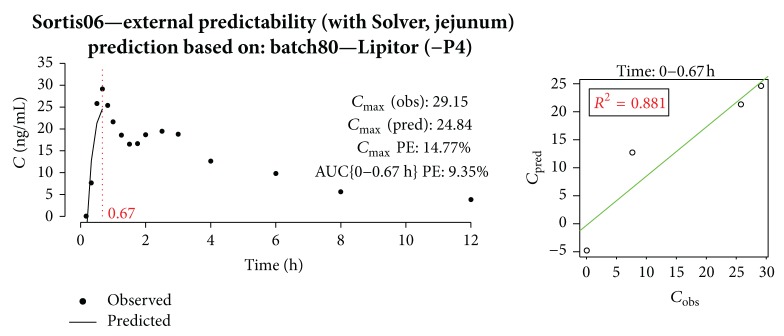
Prediction of Sortis06 profile with model built on batch80 and Lipitor.

In Figure 20, the prediction for Sortis06 is shown. Prediction errors were below 20%, which means that according to FDA guidance [15] the model is not to be rejected, but additional data should be provided in order to be fully validated.

In summary, the compartmental approach directly correlated the* in vitro* jejunal dissolution profile with the course of the plasmatic versus time profile. The logical basis for this correlation was the assumption that any dissolved portion of API* in vivo* would be immediately absorbed due to the nonlimiting permeability of ATV, which allows direct correlation of the ascending part of the dissolution and plasmatic profiles. This approach provided the best correlation options even for the combination of both formulation designs. This could be accounted for the fact that each compartment of the SI contained additional 33 mL of starting volume, and this portion of fresh medium in the jejunum provided sufficient sink-condition to allow dissolution of API without saturation of the solution during the early minutes in this compartment. The differences between the batches were thus more pronounced here than elsewhere in the apparatus.

From the correlation-development view, however, the basis for direct correlation was purely empirical. The approach with direct mapping of jejunum profiles with the PK profiles was based on the observation of profound similarity of both types of the results. Regardless of any mechanistic considerations presented above, the most appealing point here was to discard complicated convolution/deconvolution methods in favor of simple linear or nonlinear mapping of* in vitro* to the* in vivo* profiles. Thus, intravenous administration of the API was no longer necessary here, and mathematical limitations of convolution/deconvolution techniques were not anymore applicable. It should be, however, pointed out that in order to achieve such a relatively simple mathematical model, it is necessary to provide physiologically relevant dissolution results. In theory, this can be regarded as an inverse proportion between the sophistication of numerical procedures necessary for IVIVC/IVIVR and the degree of faithful representation of biological processes by the dissolution method. Therefore, the presented models demonstrating very good correlation could serve as an indirect proof of the Golem's biorelevance. Moreover, in the future, the Golem's flexibility could allow defining its critical operational parameters that could be optimized in order to achieve its maximum biorelevance in regard to the individual formulations tested. An apparatus like Golem can utilize dissolution method easily tailored to a particular formulation or API, based on the quantitative and qualitative composition, knowledge on the API's physicochemical and biopharmaceutical characteristics and other factors. An individual formulation-related dissolution protocol could thus be an answer to the industrial need for discriminative and at the same time biorelevant dissolution methods, whereas standardization could be achieved on the level of algorithmic approach to the adjustment of the dissolution protocol.

## 4. Conclusions

Dissolution of eight IR batches of ATV was tested using a novel dissolution apparatus allowing dynamic simulation of stomach and small intestine. The biorelevant conditions and observation of succeeding dissolution processes in separate compartments provided comprehensive information on the dissolution behavior expected* in vivo*. Such information and elucidation of the connections between processes happening under different conditions, which are however connected* in vivo*, are often inaccessible with the use of less complex apparatuses. The dissolution testing was performed with a basic universal dissolution method based on a faithful simulation of the GI tract. As such, it defined the main effects influencing dissolution performance of the tested batches, which is an important and often difficult step in research and development (R&D) and is a basic prerequisite for further dissolution method optimization, performed according to the characteristics of the given API and its formulation. Due to the complex nature of the presented apparatus, any modification of the dissolution method can be easily performed. In case of the correlation options, the structure of Golem apparatus allowed treating different compartments separately, adding or subtracting the measurement results for each compartment. As a result, two approaches to* in vitro*-*in vivo* correlation could be developed. The main advantage of the presented methods was no requirement for additional intravenous drug administration (simulated data for* i.v.* administration was used in first approach) which might be inaccessible in certain cases. Whole analysis with deconvolution was performed in a single *R* script and therefore results are completely reproducible. Compartmental approach can be redone in a spreadsheet and standardized according to the industrial requirements, thus presenting simple and effective tool for quick establishment of IVIVC/IVIVR based on Golem's results.

In order to fully evaluate the Golem's capabilities and the presented correlation techniques, larger dataset with greater differences in dissolution rates should be used.

The future work with the apparatus will be aimed at generating and processing of further larger datasets for further verification of the hypotheses presented in this paper.

## Figures and Tables

**Table 1:** Dissolution method description.

	Stomach	Duodenum	Jejunum	Ileum
Starting volume (mL)	30 (gastric juice) + 200 (deionized water)	33	33	33
Residence/transit time (min)	30; linear emptying	10	60	90
pH	2.4 (at 37°C)	6.5	6.6	7.4
Pepsin (mg/mL)	1.3	—	—	—
Lipase activity (U/mL)	—	70	70	70
Bile salts (mM)	—	3	3	3

**Table 2:** Intravenous administration simulation parameters.

Parameter group	Parameter	Value			Source

Physicochemical and binding	Mol. weight (g/mol)	**558.6**			[http://www.drugbank.ca/]
	log⁡*P*	**5.7**			[http://www.drugbank.ca/]
	Compound type	Monoprotic Acid			[http://www.drugbank.ca/]
	pK_a_ 1	4.330			[http://www.drugbank.ca/]
	B/P	0.610			[7]
	Fu	0.107			[8]

ADME	Enzymatic clearance				
	Pathway	p-Hydroxy	o-Hydroxy	p-Hydroxy	
	Enzyme	CYP3A4	CYP3A4	CYP2C8	
	*V*_max⁡_	**29.800**	**29.300**	**0.290**	[9]
	*K*_*m*_	**25.600**	**29.700**	**35.900**	
	fu mic	**0.662**	**0.662**	**0.662**	
	Total plasma clearance-CL (Hep)	**26.93 L/h**			[Fitted to the clinical data]

log⁡*P*—logarithm of the octanol-water partition coefficient; pK_a_—dissociation constant; B/P—blood-to-plasma partition coefficient; fu—fraction unbound in plasma; *V*_max⁡_—maximum reaction rate achieved by the system; *K*_*m*_—substrate concentration at which the reaction rate is half of *V*_max⁡_.
